# Technological innovation facilitates the practice of “three-dimensional ecology”

**DOI:** 10.1016/j.isci.2022.105767

**Published:** 2022-12-09

**Authors:** Yanwen Fu, Guangcai Xu, Yumei Li, Shang Gao, Qinghua Guo, Haitao Yang

**Affiliations:** 1 Ministry of Education Key Laboratory for Biodiversity Science and Engineering, Northeast Tiger and Leopard Biodiversity National Observation and Research Station, National Forestry and Grassland Administration Amur Tiger and Amur Leopard Monitoring and Research Center, National Forestry and Grassland Administration Key Laboratory for Conservation Ecology in Northeast Tiger and Leopard National Park, College of Life Sciences, Beijing Normal University, Beijing, China; 2 Institute of Remote Sensing and Geographic Information System, School of Earth and Space Sciences, Peking University, Beijing 100871, China; 3 Beijing GreenValley Technology Co., Ltd, Haidian District, Beijing 100091, China; 4 Key Laboratory of Animal Ecology and Conservation Biology, Institute of Zoology, Chinese Academy of Sciences, Beijing 100101, China

**Keywords:** Environmental technology, Ecology, Biological sciences

## Abstract

The development of “three-dimensional ecology” reveals refreshing phenomena and challenges us to use three-dimensional information for studying animal perception. We created a new processing framework to quantify the shielding effect using a reconstructed environmental structure. The framework achieves three objectives: 1) the observed is introduced, 2) the observed space size can be flexibly dealt with, and 3) three-dimensional attributes are assigned to the environmental structure. Our processing framework is an applicable method to “three-dimensional ecology” based on the three-dimensional attributes of physical structures. We advocate for greater emphasis on “three-dimensional ecology” to recreate realistic animal living conditions and better reveal their behaviors.

## Highlights

• A tool for quantifying shielding effects using 3D environment structures
• The terrain relief and the target size will affect the shielding effect
• The developed tool broadens the application of 3D structure in ecology

## Graphical abstract

**Figure:**
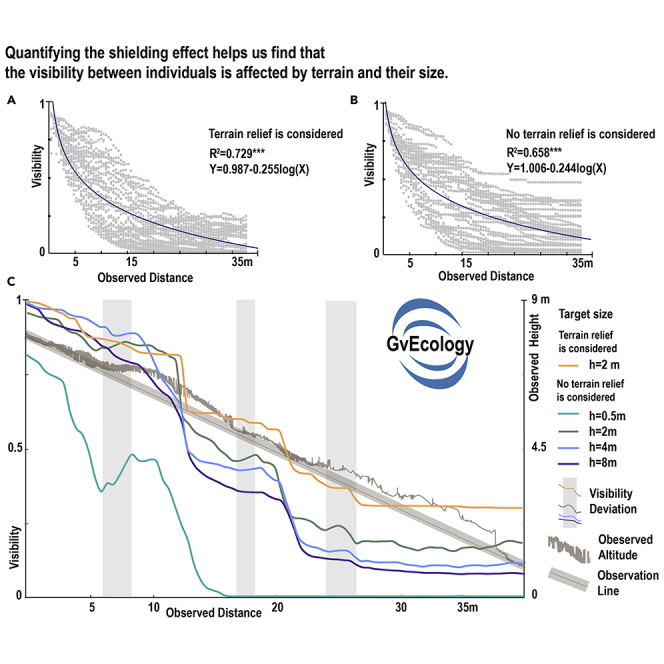

## Introduction

“Three-dimensional (3D) ecology” urges us to apply reconstructed environmental structures to ecological research.^1^ The reconstruction of environmental structure has been applied in human environments such as urban planning,^2^ surveying and mapping,^3^ powerline inspection,^4^ etc., but the extension to ecological applications has been limited. The relationship between organisms and the environment is an important topic in ecology, such as in habitat use and mediating interspecific relationships.^5^^,^^6^^,^^7^^,^^8^ Previous study based on Geographic Information System (GIS) to analyze remote sensing images in Serengeti National Park has found that lions (*Panthera leo*) tend to choose habitats with better hiding for themselves, rather than the area with more prey.^9^ However, limited by the 2D properties of remote sensing images, it is difficult to verify them in complex forest ecosystems. In recent years, with advances in 3D technology, ecologists discovered unique phenomena using reconstructed environments, such as coexistence between predators^8^ and differences in habitat selection between predators and prey mediated by environmental structures.^10^ The emergence of these exciting results stimulated our interest in the relationship between organisms and the environment within reconstructed environmental structures.^11^

Applying reconstructed environmental structures to ecological research is challenging. In the study of terrestrial ecosystems, researchers have paid attention on the shielding effect of environment structures.^12^^,^^13^^,^^14^^,^^15^ Different from the existing definition,^16^ in this study, shielding effect refers to the degree to which the environment structure occludes the object (The object refers to the observer, the observed, or even the environment because they are all research subjects). In the existing studies on terrestrial ecosystems, the shielding effect of environmental structure is reflected as visual occlusion. For example, studies on predator perches show that prey visibility increases with perch height in open habitats.^7^ African wild dogs (*Lycaon pictus*) choose relief terrain to avoid lions and lessen their risk of predation.^8^ These studies qualitatively discussed the effect of the shielding effect of the environment on the study objects. After quantifying the shielding effect, we are more interested in how it functions amongs subjects than whether it works. We want to know how the shielding effect is altered by changes in the environment’s structure, as well as the relationships between the studied objects. Researchers studying the interaction between the environment and organisms are interested in this as well.^17^^,^^18^ Although these studies have attempted to quantify the shielding effect, most of them have substantial limitations.

Whether we can quantify the shielding effect between objects determines whether we can effectively use 3D information and promote the development of “3D ecology”. Previous study has attempted to quantify the shading effect of vertical pathways.^19^ In forest, ecosystems, the quantification of horizonal pathways is also important because most organisms depend on terrain for survival and shielding in horizonal pathways^8^^,^^20^; in particular, mammals experience severe horizonal predation pressure. The quantified shielding effect between objects is based on the target detection principle,^10^ and the shielding to the observed is our concern. The development of Light Detection and Ranging (LiDAR) provides us with an opportunity to quantify the shielding to the observed because it can obtain high-precision environmental point cloud data and then reconstruct the environmental structure.^1^ Previous studies sought to quantify the observer’s perceived range by assuming the environment as the observed and the observed range as a spherical surface defined by the observer’s distance.^12^^,^^13^^,^^14^ This is appropriate when the environment is seen as being observed, and confusion arises when the observed is other organisms. For example, when the environment is the observed, we can assume that the observed area is the spherical surface around the observer. The shielding effect is characterized by calculating the occlusion of the sphere surface by the environment point cloud. However, when the organism is the observed, the environment occludes the observed, the observed area varies with the size of the organism, and it’s no longer a sphere surface. The shielding effect needs to be characterized by the occlusion of the organism by the environmental point cloud. The assumption of sphere surface limits researchers from reasonably quantifying the environmental shielding effect between organisms.

The relationship between organism and environment encompasses not just the impact of the environment on a single organism but also the impact of inter-organism interactions.^18^ When the observer and the observed are both organisms, as the observed moves, the range of the environmental point cloud forming a shielding will change as a result of the movement. The range of point cloud forming a shield is related to its spatial position, including horizontal and vertical positions, and even the size of the target. When the observed is vertically elevated, lower part of an obstacle that previously formed an occlusion may no longer form an occlusion. This is because the obstacle has 3D attributes. With the change of the position relationship between the observer and the observed, the part of the obstacle contributing to the occlusion changes. We believe that this change due to 3D attributes needs to be taken seriously in research because it is real, yet little has been reported. This suggests that the quantitative shielding effect still has a lot of room for improvement, allowing for more effective utilization of 3D information and promoting the advancement of “3D ecology”.

Here, we developed a new method to quantify the shielding effect. We give the environmental structure 3D attributes. We flexibly deal with the space size of the observed and the range of point cloud forming the shielding effect (Figure S1). Furthermore, we demonstrate the effectiveness of our method in detecting these factors. We explored the general patterns of visibility changes and the effects of terrain relief and observed space size on visibility changes in both normalized and not-normalized scenarios.

## Results

### General pattern of visibility changes

**Figure 1:**
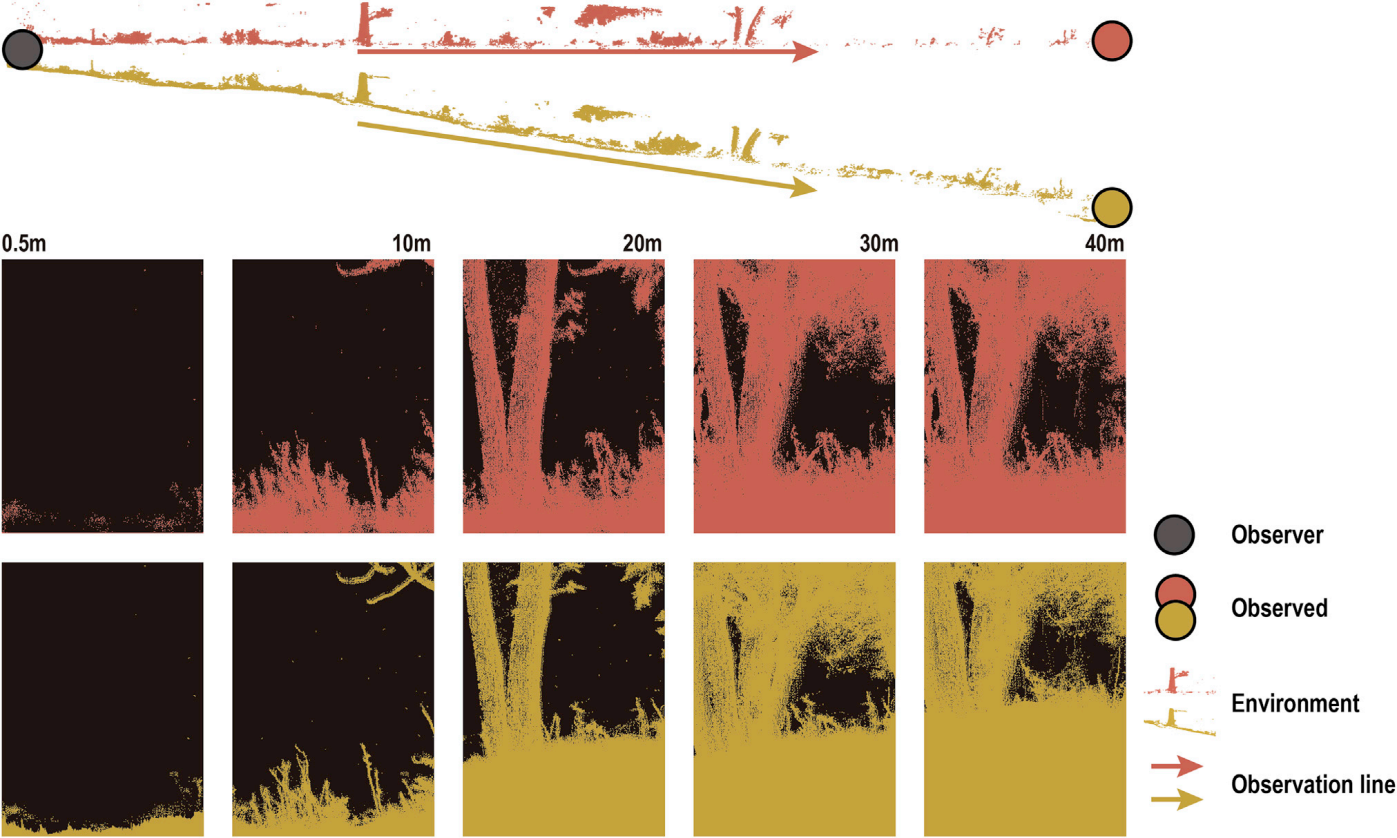
Cumulative effects of normalization and non-normalization on visibility Red represents normalization and yellow represents non-normalization. After normalization, the lowest point of the point cloud is at the same altitude, and the lowest point of the point cloud fluctuates with the terrain when the point cloud is not normalized. In both scenarios, visibility decreases as distance increases due to cumulative effects. The data came from the 80-degree direction.

Normalization of the point cloud removes the effect of terrain by standardizing the lowest points to an altitude of zero (Figure 1, Tables S1 and S2), and consequently, the cumulative effect of the point cloud on occlusion of objects in the distance is strictly enforced. However, in the non-normalized scenario, the terrain can alter the effect of the point cloud on occlusion of an object in the distance as a function of the terrain surface between the observer and observed target (Video S1).

Video S1. Normalized and non-normalized visibility changes, related to Figure 1 The data came from the 80-degree direction.

**Figure 2:**
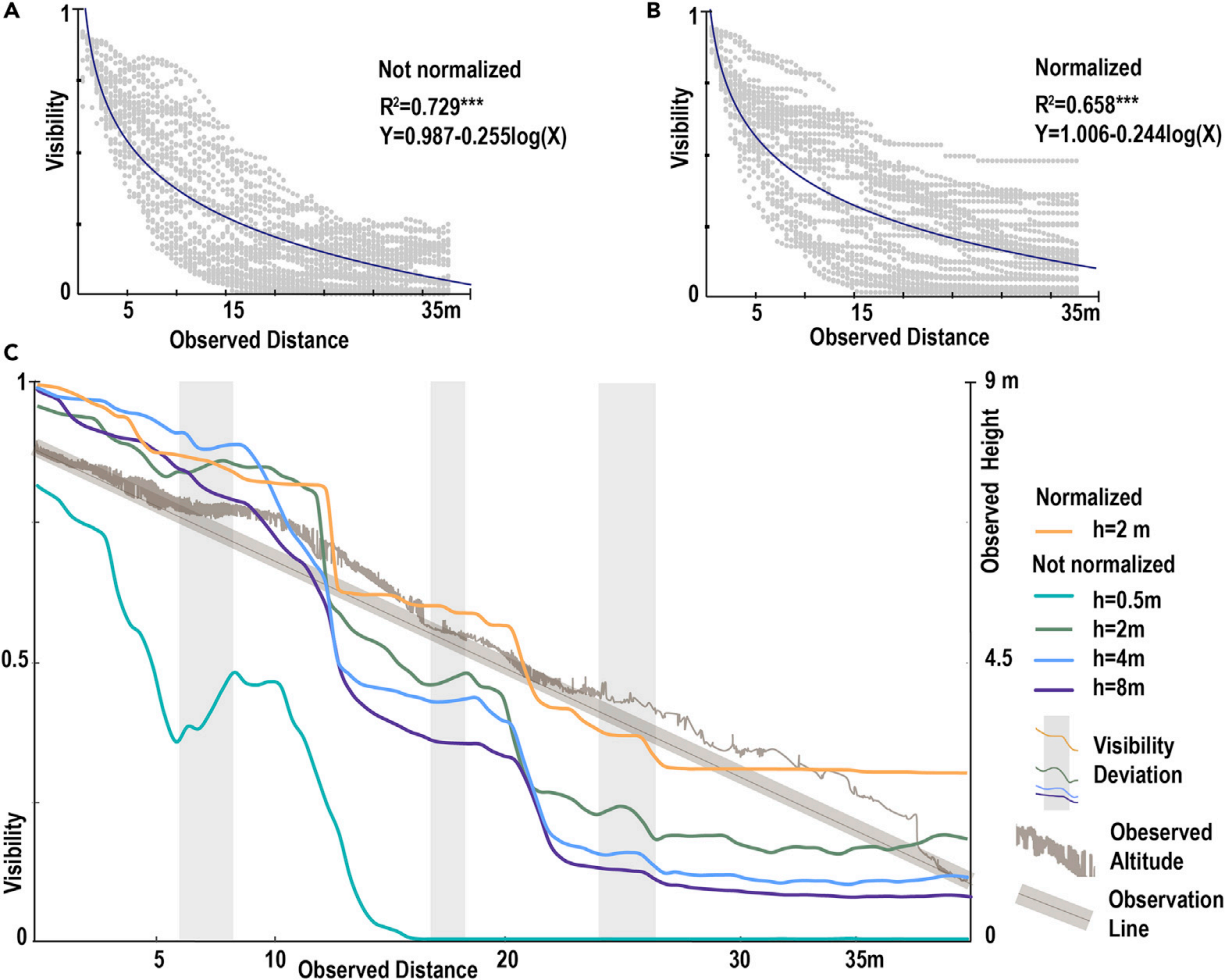
Patterns and sources of deviation in visibility (A) Variation of visibility under non-normalized scenarios. (B) Variation of visibility under normalized scenarios. In both scenarios, the relationship between visibility and distance conforms to visibility = a∗log (distance) + b mode. (C) Relationship between visibility deviation, terrain relief, and object size in the direction of 80°. In the normalized scenario, visibility does not deviate and cumulative effects are observed. In the not-normalized scenario, the visibility deviates due to the terrain relief, and the visibility increases. As the size of the object changes, the degree of visibility deviation changes. The relationship between distance and visibility was obtained by logarithmic regression fitting, ∗∗∗ represents p < 0.001.

We used curves to fit the relationship between visibility and distance and obtained a general model of visibility as a function of the distance. In both scenarios, the relationship between visibility and distance is logarithmic (normalization: visibility = 1.006–0.244 log(distance), R^2^ = 0.658∗∗∗, non-normalization: visibility = 0.987–0.255 log(distance), R^2^ = 0.729∗∗∗) (Figures 2A and 2B).

### Changes in visibility under terrain relief

When the altitude position of the target changes, we find visibility deviation: the visibility changes (either increase or decrease) as a function of terrain. Point clouds in the direction of 80° were extracted for comparison under normalized and not-normalized scenarios (Figure 2C, normalized: h = 2 m, not-normalized: h = 2 m). In the normalized scenario, visibility deviation did not occur with terrain relief, while in the not-normalized scenario, visibility deviation occurred with terrain relief.

### Changes in visibility with changes in the observed size

We found that different object sizes resulted in different visibility changes (Data S1). We also used 80-degree direction point cloud data but set different object sizes in the non-normalized scenario. We set four gradients for the object size (h = 0.5 m, h = 2 m, h = 4 m, and h = 8 m) under the assumption that the width of the object remains unchanged (Figure 2C). When h = 0.5 m, the visibility deviation was the most sensitive and decreased fast to its lowest value. With the increase of h, the visibility deviation was desensitized (Figure 2C). When h = 8 m, the terrain relief could no longer cause visibility deviation.

## Discussion

The development of “3D ecology” has been driven by technological innovation. Previously, 3D information in terrestrial ecosystems was limited by the difficulty of reconstructing the environmental structure.^1^^,^^21^ However, with the development of LiDAR technology, it is possible to obtain high-precision environmental point clouds with portable instruments.^21^ Especially, the development of ground-based LiDAR including backpack and handheld which can maintain centimeter-level accuracy to meet the needs of point cloud reconstruction.^1^^,^^10^ With the popularization of LiDAR technology and new processing frameworks based on 3D information, we are gradually able to recreate real animal worlds.

Our study systematically reports the quantification of shielding effects in “3D ecology”. We include 3D attributes of environmental structure into the analysis, providing an analytical framework for quantifying the shielding effect between organisms, including the 3D position change and size change of the observed.

In a non-normalized scenario, visibility deviation occurs with terrain relief, which indicates that it is not a random error but a real phenomenon. In ecological research, we need to seriously consider indicator anomalies produced by the terrain. In traditional ecology, we may regard these abnormal indicators as systematic errors, but in the context of “3D ecology”, these “errors” in the traditional sense are real and explorable phenomena.

Object size changes the relationship between terrain and visibility. Terrain no longer causes visibility deviation with larger objects but increases visibility deviation with smaller objects. This is consistent with existing research showing wild dogs use terrain to coexist with predators in the blind spot of a lion’s vision.^8^ Under the same terrain background, objects of different sizes will form different visibility conditions. For the observer and the observed, this principle provides better hiding conditions for small objects and forms a “visual shelter”. This may be an underappreciated mechanism for species coexistence.

Understanding how animals use a landscape in response to their habitat composition is a crucial question.^22^ Ecologists generally choose resource selection functions (RSFs) or step selection functions (SSFs) to investigate this problem. But the debate between the two models persists. It is unreasonable for RSF to assume that all spatial pixels have the same accessibility.^23^ This is manifested not only in the physical inaccessibility of time and space but also in the invisibility of vision (Figure 2). The SSF has made improvements to this problem. At a given time interval (usually the sampling interval),^24^ an animal’s potential accessibility of displacement to a location is based on habitat composition in the neighborhood of the animal’s current position.^25^ Even so, there is still a question of whether the sampling interval can determine the boundary of an analyzed pixel in each step. Our results show that the visibility of animals is affected by the environment, and the visibility decreases to a minimum over a distance threshold. This means that animals cannot fully perceive the environment beyond the boundaries of visibility.^20^ The distance threshold is constantly changing, influenced by the environment, the object size, and the terrain. In the case of visibility alone, that means that at every step of the SSF, the analysis boundary changes depending on the environment, the object size, and the terrain. The range of animals' perception of the environment is also closely related to the width of the animal protection corridor.^26^ We believe that vision-based animal perception of the environment should be taken into account in the application of SSF for habitat or corridor selection.

The “landscape of fear” is an important concept in ecology, integrating behavioral, population, and community responses to predation and providing a central organizing principle for the study of predator-prey dynamics on heterogeneous landscapes.^27^ In the research framework of the landscape of fear, landscape structure has basic functional attributes, shapes visibility, detection, and movement before and during a predator-prey encounter. But quantifying risk is messy because the animals' perception of risk is based on subjective assumptions made by researchers.^28^ This leads to confusing conclusions. In the process of quantifying the shielding effect, we found that visibility could represent exposure and easily quantify risk. This is an opportunity to break subjective assumptions about how animals perceive risk. For the “landscape of fear”, on the basis of quantifying risk, there is definite data support to establish how the relationship between animals and the environment is mediated by risk.

### Limitations of the study

We did not include the imaging ability of animals’ eyes in the quantification process because different animals have different visual imaging abilities, and relevant studies need to be carried out.^29^ Nevertheless, we still believe that differences in visual imaging ability play an important role in quantifying vision. Therefore, we reserved interfaces for incorporating visual imaging ability into the analysis. We generated visual images. Their advantage is that they cannot only obtain information such as whether or not there is occlusion but also obtain information about the size of the occlusion, which means that it can be graphically processed based on the ability of visual imaging to reflect the clarity of objects for animals.

### Conclusions

Our processing framework provides a feasible solution for exploring ecological problems in a 3D environment. We also highlight the importance of using 3D information effectively. At the same time, the method based on visual quantitative development can provide innovative solutions for other ecological fields and promote cross integration in ecology. This also reminds us of the importance of reconstructing the real environmental structure for ecology, and 3D ecology should attract our attention.

## STAR★Methods

### Key resources table

**Table:** 

REAGENT or RESOURCE	SOURCE	IDENTIFIER
**Software and algorithms**		

Lidar360	Beijing GreenValley Technology Co., Ltd	https://greenvalleyintl.com/LiDAR360/
GvEcology	Beijing GreenValley Technology Co., Ltd	https://pan.bnu.edu.cn/l/LFhCXC
The original point cloud data of normalized and non-normalized scenarios	This manuscript	https://pan.bnu.edu.cn/l/0FgZFq
LiPod	Beijing GreenValley Technology Co., Ltd	https://greenvalleyintl.com/LiPod/
IBM SPSS Statistics 26	IBM Corp. in Armonk, NY	https://www.ibm.com/cn-zh/spss?lnk=flatitem

### Resource availability

#### Lead contact

Further information and requests for resources and reagents should be directed to and will be fulfilled by the lead contact, Haitao Yang (yht90h@pku.edu.cn).

#### Materials availability

The original point cloud data of normalized and non-normalized scenarios are available at https://pan.bnu.edu.cn/l/0FgZFq. GvEcology is available at https://pan.bnu.edu.cn/l/LFhCXC. LiDAR360 is available at https://greenvalleyintl.com/LiDAR360/. GvEcology runs on LiDAR360, when running GvEcology for the first time, first, you need to install LiDAR360 and second, apply for a use license according to the website (https://greenvalleyintl.com/LiDAR360/) prompts. Third, click GvEcology.exe to run the graphical interface. Fourth, input parameters as defined below, and run the program.

### Method details

We developed GvEcology, an ecological point cloud processing program that quantifies shielding effect (Normalized and not normalized scenarios), based on the platform of LiDAR360 5.2 (https://greenvalleyintl.com/LiDAR360/). The shielding effect of the physical structure formed by the point cloud on the observed is represented by the visibility. The point cloud is projected to the observed along the observer’s line of sight, and the observed is pixelated by us. The pixels occupied by the point cloud represent the shielding effect of the environment, and the unoccupied pixels represent exposure. Visibility is calculated based on the exposed area. There is no special requirement for point cloud acquisition equipment in our program. General application terrestrial LiDAR remote sensing (TLS) (e.g., VZ400, VZ400I, Lipod backpack equipment and handheld equipment) can be used as input point cloud acquisition methods. However, when collecting point cloud data, the observer position needs to be highlighted for identification in the point cloud.

We simplify the observed by using the minimum enclosing rectangle to represent the size of the observed body, excluding the tail. The height of the observed body is denoted by h and the width by w. The minimum enclosing rectangle are placed perpendicular to the ground to simulate the posture of an organism as it moves in three dimensions. The sampling of observer positions can meet different research needs. It allows the researcher to collect the visibility index of the observed (θ, i) at different distance gradients (i) in all directions (θ) within a custom circular area near the observer. Program input indicators include w, h, start Angle, end Angle, angle step size, farthest sampling distance, distance step size, and resolution.

#### Normalized analysis framework

##### Pretreatment

In the normalization scenario, the ground point identification is firstly carried out for the point cloud data obtained, and then the point cloud data is normalized according to the identified ground points. The ground point height after normalization is 0 m. Since the ground occlusion is closed but the ground points are sparse, it cannot be guaranteed that every pixel blocked by the ground points will be occupied, so the ground points need to be encrypted during interpolation processing. Then, the corresponding coordinates in the point cloud are identified according to the observer’s position (x0, y0, z0). The analysis was based on LiDAR360 5.2 (Beijing GreenValley Technology Co., Ltd).

##### Determination of projection boundary

We place the point cloud in a 3D coordinate system and use six straight lines to frame the range of the point cloud. Using the θ, i, w and h, we determine six boundaries (Table S1), including x1 and x2 on the X axis, y1 and y2 on the Y axis, and z1 and z2 on the z axis. In the normalized scenario, z1 is equal to 0, z2 is equal to h. The analysis was based on GvEcology with a normalization module (Beijing GreenValley Technology Co., Ltd).

The range of point clouds projected to the minimum enclosing rectangle (Figures S1C and S1D, Table S1):

$$p_{\theta ,i}=\left\{ \begin{array}{l} y1<y<y2\\ x1<x<x2\;\\ 0<z<h \end{array}\right.$$

$p_{\theta ,i}$ is the minimum peripheral cube of the point cloud used for projection.

Due to the normalization of the point cloud pretreatment, the z value of the point cloud is always limited in the range [0,h].

##### Determination of projection direction

The direction of the vector $\vec{z_{\text{ground}\;\text{point}\;\text{of}\;\text{observer}\;\text{coordinates}}z_{\text{ground}\;\text{point}\;\text{of}\;\left(\theta ,i\right)}}$ is the projection direction (Figure S1D). Since the point cloud is normalized, the vector direction changes only in the (x, y) plane.

Let the original point cloud coordinate be (x, y, z).

The point cloud coordinates processed by projection are (x_p_, y_p_, z_p_) (Table S2).

After projecting the point cloud to the minimum enclosing rectangle, the mapping of (x_p_, y_p_, z_p_) in the plane (x, y) falls on the line (x1, x2, y1, y2) represented by the boundary of the point cloud farthest from the observer (Table S1). Given the line and the point, the perpendicular foot of the point to the line is (x_p_, y_p_) (Table S2). In the normalized scenario, z_p_ is equal to z. The analysis was based on GvEcology with a normalization module (Beijing GreenValley Technology Co., Ltd).

##### Images generated

After projection transformation, the point cloud plane is formed in the position of the minimum enclosing rectangle, and the visual image is formed according to the preset w, h and resolution. Based on the resolution, a binary classification is carried out on the image, where the pixel value occupied by the point is 1, and the pixel value not occupied by the point is 0. The analysis was based on GvEcology (Beijing GreenValley Technology Co., Ltd).

$$The\;pixel\;width\;of\;the\;image=\frac{w}{resolution\;}$$

$$The\;pixel\;height\;of\;the\;image=\frac{h}{resolution\;}$$

##### Calculation of visibility

$$V_{\theta ,i}=\frac{N-n_{\theta ,i}}{N}*100\%$$

N=w∗h

Vθ,i is the visibility of observed (θ, i), N is the area of the minimum enclosing rectangle.

nθ, i is the area where the observed is obscured by the point cloud. The analysis was based on GvEcology (Beijing GreenValley Technology Co., Ltd)

#### Not normalized analysis framework

##### Pretreatment

The difference from the normalized scenario is that in the not normalized scenario, the point cloud does not undergo normalized preprocessing, which means that the altitude of the ground points reflects the real terrain relief (Figures S1E and S1F).

##### Determination of projection boundary

Compared to the normalized scenario, in the not normalized scenario the the z value is not necessarily in the range of 0 -h, because the relief of the terrain is taken into account. With the relief of the terrain, the upper and lower boundary of the point cloud on the z axis is parallel to the projection direction $\vec{z_{\text{ground}\;\text{point}\;\text{of}\;\text{observer}\;\text{coordinates}}z_{\text{ground}\;\text{point}\;\text{of}\;\left(\theta ,i\right)}}$ of the point cloud and intersects with the upper and lower boundary of the minimum enclosing rectangle in the (x, z) plane. The analysis was based on GvEcology without a normalization module (Beijing GreenValley Technology Co., Ltd). The range of point clouds projected to the minimum enclosing rectangle:

$$p_{\theta ,i}=\left\{ \begin{array}{l} y1<y<y2\\ x1<x<x2\\ z1<z<z2 \end{array}\right.$$

z1=tan(Ω)∗x

z2=tan(Ω)∗x+h

$$\Omega =\arctan\left(\frac{z_{\theta ,i}-z0}{i}\right)$$

Ω∈(−90°,90°)

$p_{\theta ,i}$ is the minimum peripheral cube of the point cloud used for projection.

##### Determination of projection direction

After projecting the point cloud to the minimum enclosing rectangle, the mapping of (x_p_, y_p_, z_p_) in the plane (x, y) also falls on the line (x1, x2, y1, y2) represented by the boundary of the point cloud farthest from the observer (Table S1). Given the line and the point, the perpendicular foot of the point to the line is (x_p_, y_p_) (Table S2). Compared to the normalized scenario, in the not normalized scenario, z_p_ varies according to the terrain relief and is not necessarily equal to z (Figure S1F). First, we calculate the distance between the point and the line, then, calculate the elevation change of the minimum enclosing rectangle due to the relief of the terrain by trigonometric function, correct the z value, and get the final z_p_. The analysis was based on GvEcology without a normalization module (Beijing GreenValley Technology Co., Ltd).

$$z_{p}=z+\tan\left(\Omega \right)\times distance\left(point-line\right)$$

##### Images generated

Same as the normalized scenario.

##### Calculation of visibility

Same as the normalized scenario.

#### Point cloud collection and processing

We use LiPod (Beijing GreenValley Technology Co., Ltd) to collect point cloud data. Using a VLP16 radar sensor, the LiPod measures with a relative accuracy of ≤3 cm and an absolute accuracy of ≤5 cm, with a vertical field of view Angle of ±15°, a horizontal field of view Angle of 360°, and a maximum measurement distance of 100 m. We set up 10 sites within the sampling range of 90 m × 90 m to collect data separately in order to obtain the complete point cloud with high accuracy. The collected data were registered and concatenated using LiDAR360 ^10^. The forest type in the sampling area was broadleaved forest, with an average tree height of 5.05 m and an average DBH of 0.36m. Calculated by LiDAR360.

### Quantification and statistical analysis

We calculated visibility using GvEcology. Parameter Settings are as follows. The coordinates of the central point were set as (0.246, 0.088, −1.148) in the not normalization scenario, and (0.246, 0.088, 0) in the normalization scenario. The starting angle was 10°, the ending angle was 360°; the angle step interval was 10°, the farthest sampling distance was 40 m, the equal rectangle width (w) was 1.5 m, the height (h) was set four times - 0.5 m, 2 m, 4 m and 8 m, the distance step length was 0.5 m, and the equivalent rectangle resolution was 0.005 (Figure 1). In each scenario, we obtained 36 directions with 80 equal rectangles in each direction for a total of 2880 sets of distance-visibility data (Data S1). The relationship between distance and visibility was obtained by logarithmic regression fitting, ∗∗∗ represents p < 0.001. Used IBM SPSS Statistics 26 (IBM Corp. in Armonk, NY).

## Data Availability

• The original point cloud data of normalized and non-normalized scenarios has been deposited at Cloud Drive, and URLs are listed in the key resources table. • This paper does not report original code. The code runner GvEcology can be found at key resources table. • Any additional information required to reanalyze the data reported in this paper is available from the lead contact upon request.

## Supplemental information

Document S1. Figure S1 and Tables S1 and S2

Data S1. Data sources of Figure 2, related to Figure 2

## References

[1] Davies A.B., Asner G.P. (2014). Advances in animal ecology from 3D-LiDAR ecosystem mapping. Trends Ecol. Evol..

[2] Hu T., Wei D., Su Y., Wang X., Zhang J., Sun X., Liu Y., Guo Q. (2022). Quantifying the shape of urban street trees and evaluating its influence on their aesthetic functions based on mobile lidar data. ISPRS J. Photogrammetry Remote Sens..

[3] Cong Y., Chen C., Yang B., Li J., Wu W., Li Y., Yang Y. (2022). 3D-CSTM: a 3D continuous spatio-temporal mapping method. ISPRS J. Photogrammetry Remote Sens..

[4] Guan H., Sun X., Su Y., Hu T., Wang H., Wang H., Peng C., Guo Q. (2021). UAV-lidar aids automatic intelligent powerline inspection. Int. J. Electr. Power Energy Syst..

[5] Kareiva P. (1987). Habitat fragmentation and the stability of predator–prey interactions. Nature.

[6] Warren M.S., Hill J.K., Thomas J.A., Asher J., Fox R., Huntley B., Roy D.B., Telfer M.G., Jeffcoate S., Harding P. (2001). Rapid responses of British butterflies to opposing forces of climate and habitat change. Nature.

[7] Andersson M., Wallander J., Isaksson D. (2009). Predator perches: a visual search perspective. Funct. Ecol..

[8] Davies A.B., Tambling C.J., Marneweck D.G., Ranc N., Druce D.J., Cromsigt J.P.G.M., le Roux E., Asner G.P. (2021). Spatial heterogeneity facilitates carnivore coexistence. Ecology.

[9] Hopcraft J.G.C., Sinclair A.R.E., Packer C. (2005). Planning for success: Serengeti lions seek prey accessibility rather than abundance. J. Anim. Ecol..

[10] Fu Y., Xu G., Gao S., Feng L., Guo Q., Yang H. (2022). LiDAR reveals the process of vision-mediated predator - prey relationships. Rem. Sens..

[11] Brito-Morales I., Schoeman D.S., Everett J.D., Klein C.J., Dunn D.C., García Molinos J., Burrows M.T., Buenafe K.C.V., Dominguez R.M., Possingham H.P., Richardson A.J. (2022). Towards climate-smart, three-dimensional protected areas for biodiversity conservation in the high seas. Nat. Clim. Change.

[12] Lecigne B., Eitel J.U.H., Rachlow J.L. (2020). viewshed3d: an r package for quantifying 3D visibility using terrestrial lidar data. Methods Ecol. Evol..

[13] Aben J., Signer J., Heiskanen J., Pellikka P., Travis J.M.J. (2021). What you see is where you go: visibility influences movement decisions of a forest bird navigating a three-dimensional-structured matrix. Biol. Lett..

[14] Aben J., Pellikka P., Travis J.M.J. (2018). A call for viewshed ecology: advancing our understanding of the ecology of information through viewshed analysis. Methods Ecol. Evol..

[15] Stein R.M., Lecigne B., Eitel J.U.H., Johnson T.R., McGowan C., Rachlow J.L. (2022). Vegetation and vantage point influence visibility across diverse ecosystems: implications for animal ecology. Front. Ecol. Evol..

[16] Grøntvedt R.N., Kristoffersen A.B., Jansen P.A. (2018). Reduced exposure of farmed salmon to salmon louse (Lepeophtheirus salmonis L.) infestation by use of plankton nets: estimating the shielding effect. Aquaculture.

[17] Zhong Z., Li G., Sanders D., Wang D., Holt R.D., Zhang Z. (2022). A rodent herbivore reduces its predation risk through ecosystem engineering. Curr. Biol..

[18] Zhang H., Bearup D., Nijs I., Wang S., Barabás G., Tao Y., Liao J. (2021). Dispersal network heterogeneity promotes species coexistence in hierarchical competitive communities. Ecol. Lett..

[19] Ashcroft M.B., Gollan J.R., Ramp D. (2014). Creating vegetation density profiles for a diverse range of ecological habitats using terrestrial laser scanning. Methods Ecol. Evol..

[20] Olden J.D., Schooley R.L., Monroe J.B., Poff N.L. (2004). Context-dependent perceptual ranges and their relevance to animal movements in landscapes. J. Anim. Ecol..

[21] Vierling K.T., Vierling L.A., Gould W.A., Martinuzzi S., Clawges R.M. (2008). Lidar: shedding new light on habitat characterization and modeling. Front. Ecol. Environ..

[22] Michelot T., Blackwell P.G., Matthiopoulos J. (2019). Linking resource selection and step selection models for habitat preferences in animals. Ecology.

[23] Matthiopoulos J. (2003). The use of space by animals as a function of accessibility and preference. Ecol. Model..

[24] Gautestad A.O., Loe L.E., Mysterud A. (2013). Inferring spatial memory and spatiotemporal scaling from GPS data: comparing red deer Cervus elaphus movements with simulation models. J. Anim. Ecol..

[25] Forester J.D., Im H.K., Rathouz P.J. (2009). Accounting for animal movement in estimation of resource selection functions: sampling and data analysis. Ecology.

[26] Scharf A.K., Belant J.L., Beyer D.E., Wikelski M., Safi K. (2018). Habitat suitability does not capture the essence of animal-defined corridors. Mov. Ecol..

[27] Gaynor K.M., Brown J.S., Middleton A.D., Power M.E., Brashares J.S. (2019). Landscapes of fear: spatial patterns of risk perception and response. Trends Ecol. Evol..

[28] Peers M.J.L., Majchrzak Y.N., Neilson E., Lamb C.T., Hämäläinen A., Haines J.A., Garland L., Doran-Myers D., Broadley K., Boonstra R., Boutin S. (2018). Quantifying fear effects on prey demography in nature. Ecology.

[29] Banks M.S., Sprague W.W., Schmoll J., Parnell J.A.Q., Love G.D. (2015). Why do animal eyes have pupils of different shapes?. Sci. Adv..

