# Supplementary material for: Technological innovation facilitates the practice of “three-dimensional ecology”
Source: iScience. 2022 Dec 9;26(1):105767. doi: 10.1016/j.isci.2022.105767 (PMC9800287; doi:10.1016/j.isci.2022.105767)
Supplement: Document S1. Figure S1 and Tables S1 and S2 [file mmc1.pdf]

iScience, Volume *26*

Supplemental information

Technological innovation facilitates the practice of “three-dimensional ecology”

Yanwen Fu, Guangcai Xu, Yumei Li, Shang Gao, Qinghua Guo, and Haitao Yang

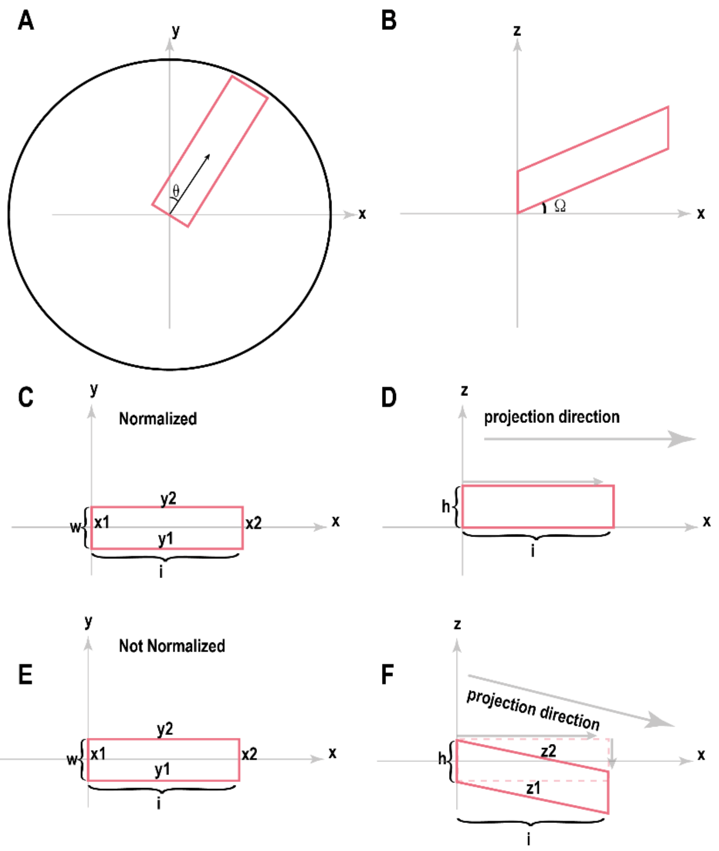

Figure. S1 Determination of projection boundary and projection direction. The red box represents the point cloud forming the occlusion, defined by the size (high=h, wide=w) of the observed. We use the minimum enclosing rectangle to represent the observed, and the line of sight between the observer and the observed is the projection direction. When the point cloud is projected to the minimum enclosing rectangle along the projection direction, a point cloud grid is formed within the equivalent rectangle, and the pixels occupied by the point cloud represent that the observed is blocked by the environmental structure. (A-B) shows ɵ and Ω. (C-D) shows normalization and (E-F) shows not normalization scenarios. Related to STAR Methods.

Table S1 The range of point clouds projected to the minimum enclosing rectangle under normalized scene. We simplify the observed by using the minimum enclosing rectangle to represent the size of the observed body, excluding the tail. Related to STAR Methods.

Value of direction (θ)	The expression for line x1	The expression for line x2	The expression for line y1	The expression for line y2
0<θ<90	$x1 = \frac{y}{-\tan(\theta)}$	$x2 = \frac{y - {}^{i}/_{\cos(\theta)}}{-\tan(\theta)}$	$y1 = \tan(90-\theta) * x - \frac{w/2}{\sin(\theta)}$	$y2 = \tan(90-\theta) * x + \frac{w/2}{\sin(\theta)}$
θ=90	$x1 = 0$	$x2 = i$	$y1 = -\frac{w}{2}$	$y2 = \frac{w}{2}$
90<θ<180	$x1 = \frac{y}{\tan(180-\theta)}$	$x2 = \frac{y + {}^{i}/_{\cos(180-\theta)}}{\tan(180-\theta)}$	$y1 = -\tan(\theta-90) * x - \frac{w/2}{\sin(180-\theta)}$	$y2 = -\tan(\theta-90) * x + \frac{w/2}{\sin(180-\theta)}$
θ=180	$x1 = -\frac{w}{2}$	$x2 = \frac{w}{2}$	$y1 = 0$	$y2 = -i$
180<θ<270	$x1 = \frac{y + {}^{i}/_{\cos(\theta-180)}}{-\tan(\theta-180)}$	$x2 = \frac{y}{-\tan(\theta-180)}$	$y1 = \tan(270-\theta) * x - \frac{w/2}{\cos(270-\theta)}$	$y2 = \tan(270-\theta) * x + \frac{w/2}{\cos(270-\theta)}$
θ=270	$x1 = 0$	$x2 = -i$	$y1 = -\frac{w}{2}$	$y2 = \frac{w}{2}$
270<θ<360	$x1 = \frac{y - {}^{i}/_{\cos(360-\theta)}}{\tan(360-\theta)}$	$x2 = \frac{y}{\tan(360-\theta)}$	$y1 = -\tan(360-\theta) * x - \frac{w/2}{\sin(360-\theta)}$	$y2 = -\tan(360-\theta) * x + \frac{w/2}{\sin(360-\theta)}$
θ=360	$x1 = -\frac{w}{2}$	$x2 = \frac{w}{2}$	$y1 = 0$	$y2 = i$

Table S2 Mathematical expressions of (xp, yp, zp) under the normalized scenarios. The original point cloud coordinates are (x, y, z). The point cloud coordinates processed by projection are (xp, yp, zp). Related to STAR Methods.

Value of direction (θ)	The line	x_p	y_p	z_p
0<θ<90	$l = x2$	$x_p = \frac{{\frac{1}{\tan(\theta)}}^2 * x - \frac{1}{\tan(\theta)} * y + \frac{i}{\cos(\theta) * \tan(\theta)}}{{\frac{1}{\tan(\theta)}}^2}$	$y_p = \frac{y - \frac{x}{\tan(\theta)} + \frac{i}{\cos(\theta) * \tan(\theta)^2}}{{\frac{1}{\tan(\theta)}}^2}$	z
0–90	$l–x2$	$x_p = i$	$y_p = y$	z
90<θ<180	$l–x2$	$x_p = \frac{{\frac{1}{\tan(180-\theta)}}^2 * x + \frac{1}{\tan(180-\theta)} * y + \frac{i}{\cos(180-\theta) * \tan(180-\theta)}}{{\frac{1}{\tan(180-\theta)}}^2}$	$y_p = \frac{y + \frac{1}{\tan(180-\theta)} * x - \frac{i}{\cos(180-\theta) * \tan(180-\theta)^2}}{{\frac{1}{\tan(180-\theta)}}^2}$	z
θ=180	$l=y2$	$x_p = x$	$y_p = -i$	z
180<0<270	$l–x1$	$x_p = \frac{{\frac{1}{\tan(\theta-180)}}^2 * x - \frac{1}{\tan(\theta-180)} * y - \frac{i}{\cos(\theta-180) * \tan(\theta-180)}}{{\frac{1}{\tan(\theta-180)}}^2}$	$y_p = \frac{y - \frac{1}{\tan(\theta-180)} * x - \frac{i}{\cos(\theta-180) * \tan(\theta-180)^2}}{{\frac{1}{\tan(\theta-180)}}^2}$	z
θ=270	$l–x2$	$x_p = -i$	$y_p = y$	z
270<θ<360	$l=x1$	$x_p = \frac{{\frac{1}{\tan(360-\theta)}}^2 * x + \frac{1}{\tan(360-\theta)} * y - \frac{i}{\cos(360-\theta) * \tan(360-\theta)}}{{\frac{1}{\tan(360-\theta)}}^2}$	$y_p = \frac{y + \frac{1}{\tan(360-\theta)} * x + \frac{i}{\cos(360-\theta) * \tan(360-\theta)^2}}{{\frac{1}{\tan(360-\theta)}}^2}$	z
0–360	$l–y2$	$x_p = x$	$y_p = i$	z
